# Ultra-high-resolution synchrotron phase-contrast CT enables microstructural pulmonary imaging at clinical dose levels

**DOI:** 10.1186/s12931-026-03561-1

**Published:** 2026-02-11

**Authors:** Claudia Victoria Benke, Johanna Reiser, Lorenzo D’Amico, Md Motiur Rahman Sagar, Nicola Sodini, Diego Dreossi, Adriano Contillo, Elena Longo, Marko Prašek, Stephan Stern, Elisa Baratella, Maria Assunta Cova, Marco Confalonieri, Paola Confalonieri, Fabrizio Zanconati, Stefano D’Errico, Davide Radaelli, Tommaso Bruscagin, Deborah Bonazza, Sam Bayat, Philipp Nolte, Lavinia Neubert, Christina Petzold-Mügge, Christopher Werlein, Lars Knudsen, Hoen-oh Shin, Jan-Christopher Kamp, Ali Seif Amir Hosseini, Willi Linus Wagner, Mark Oliver Wielpütz, Jürgen Biederer, Hans-Ulrich Kauczor, Frauke Alves, Giuliana Tromba, Christian Dullin

**Affiliations:** 1 Department of Diagnostic and Interventional Radiology, Heidelberg University Hospital, Heidelberg, Germany; 2 Translational Lung Research Center Heidelberg (TLRC), German Center for Lung Research (DZL), Heidelberg, Germany; 3 School of Physics and Astronomy, Monash University, Clayton, Australia; 4 Elettra-Sincrotrone Trieste S.C.p.A, Basovizza, Italy; 5 European Molecular Biology Laboratory (EMBL), Hamburg Unit c/o DESY, Hamburg, Germany; 6 Translational Molecular Imaging, Max-Planck-Institute for Multidisciplinary Sciences, Goettingen, Germany; 7 X-Spectrum GmbH, Hamburg, Germany; 8 Department of Radiology, Hospital of Cattinara, University of Trieste, Trieste, Italy; 9 Department of Pulmonology, Hospital of Cattinara, University of Trieste, Trieste, Italy; 10 Department of Medical Surgical and Health Sciences, Hospital of Cattinara, University of Trieste, Trieste, Italy; 11 Inserm UA07 STROBE Laboratory, Université Grenoble Alpes, Grenoble, France; 12 Faculty of Engineering and Health, University of Applied Sciences and Arts Goettingen, Goettingen, Germany; 13 Biomedical Research in Endstage and Obstructive Lung Disease Hannover (BREATH), German Center for Lung Research (DZL), Hannover, Germany; 14 Institute of Pathology, Hannover Medical School, Hannover, Germany; 15 Institute of Functional and Applied Anatomy, Hannover Medical School, Hannover, Germany; 16 Institute of Diagnostic and Interventional Radiology, Hannover Medical School, Hannover, Germany; 17 Department of Respiratory Medicine and Infectious Diseases, Hannover Medical School, Hannover, Germany; 18 Department of Clinical and Interventional Radiology, University Medical Center Goettingen, Goettingen, Germany; 19 Diagnostic Radiology and Neuroradiology, University Medicine Greifswald, Greifswald, Germany; 20 Faculty of Medicine, Kiel University, Kiel, Germany; 21 Faculty of Medicine and Life Sciences, University of Latvia, Riga, Latvia; 22 Department of Haematology and Medical Oncology, University Medical Center Goettingen, Goettingen, Germany

**Keywords:** Lung phantom, Propagation-based imaging, Phase-contrast CT, Synchrotron, Lung diseases

## Abstract

**Background:**

Ultra-high-resolution propagation-based synchrotron phase-contrast CT is an emerging technique for lung imaging. However, its feasibility and diagnostic potential at radiation doses comparable to those used in standard clinical procedures has yet to be established. This study aims to evaluate the performance of phase-contrast CT in comparison with state-of-the-art high-resolution multislice CT and bronchoscopy, and to validate its diagnostic accuracy histologically using porcine and, for the first time, human lung specimens.

**Methods:**

Phase-contrast CT experiments were conducted at the Italian synchrotron using lung specimens mounted in a custom-made anthropomorphic chest phantom. Imaging utilized two photon-counting detectors under various acquisition settings, followed by artificial intelligence-based denoising. Sequential imaging by phase-contrast CT, multislice CT, and bronchoscopy was performed prior to formaldehyde vapor fixation and histological dissection. Image quality was assessed quantitatively (contrast-to-noise ratio, edge sharpness, power spectra) and qualitatively via radiological scoring across 14 criteria.

**Results:**

Phase-contrast CT achieved effective pixel sizes of 0.067 mm (Hydra detector) and 0.038 mm (LAMBDA detector), at radiation doses near full-dose multislice CT (***≈*** 12 mGy). Denoising improved contrast without major loss of edge sharpness. Radiological scoring showed phase-contrast CT outperformed multislice CT in visualizing peripheral airways and fine parenchymal structures. Histological validation confirmed imaging accuracy. Limitations from source spot size (***≈*** 200 μm) were noted but did not prevent significant diagnostic improvements.

**Conclusions:**

Phase-contrast CT, combined with artificial intelligence-based denoising, offers detailed, non-invasive imaging of lung microstructures at clinically relevant radiation doses. It complements multislice CT, holds potential for clinical adoption in advanced pulmonary diagnostics, and may reduce reliance on invasive biopsies.

**Supplementary Information:**

The online version contains supplementary material available at 10.1186/s12931-026-03561-1.

## Introduction

Early and accurate diagnosis of lung diseases, as well as monitoring progression and treatment response, require high-quality imaging. However, lung microstructures remain difficult to visualize due to the organ’s deep location, low tissue-to-air ratio, and continuous movement from breathing and heartbeats. Current tools include bronchoscopy, multislice computed tomography (MSCT), and, in younger patients, magnetic resonance imaging (MRI) [1]. Bronchoscopy offers high-resolution images but is limited to central and medium-sized airways [2]. MSCT enables whole-lung coverage with 0.5 × 0.5 × 0.5 mm³ resolution [3]. However, this cannot resolve fine alveolar structures, often leading to ambiguous terms such as “honeycombing” or “ground-glass opacity” [4, 5]. Even photon-counting CT, reaching ~ 0.2 mm³ [6], cannot depict terminal bronchioles or intralobular septa [7, 8].

Propagation-based imaging (PBI), a phase-sensitive X-ray technique, overcomes these limits by exploiting monochr`omatic X-rays, photon-counting detectors, and long propagation distances [9]. PBI enables high soft-tissue contrast and spatial resolution below 0.1 mm [10], at radiation doses comparable to MSCT [11].

Although currently restricted to synchrotron facilities [11], with compact light sources representing a potential future alternative [12], ultra-high-resolution propagation-based synchrotron phase-contrast computed tomography (PBI-CT) is advancing toward clinical application. This progress is driven by the SYRMEP-LS (SYnchrotron Radiation for MEdical Physics – Life Science) upgrade at the Italian synchrotron, in partnership with the Hospital of Cattinara, Euro-BioImaging, and the German Center for Lung Research. Planned for 2028, this system aims to provide second-level diagnostics for inconclusive MSCT cases, supporting clinical decision-making by improving noninvasive lesion characterization and potentially reducing the number of invasive biopsies performed solely for diagnostic clarification [13].

Since 2018, substantial technical progress has been achieved. This includes initial feasibility tests [14], development of a heavy-specimen sample stage [15], optimizing large sample-to-detector distances [11], construction of an anthropomorphic chest phantom [16], and establishment of a lung fixation protocol using formaldehyde (FA) vapor [16]. In this study, we extend these advances by testing two photon-counting detectors (LAMBDA and Hydra) and artificial intelligence (AI)-based denoising. We correlate PBI-CT with MSCT, bronchoscopy, and histology in the same lung specimen. Crucially, we present the first results on human lungs imaged within the phantom, marking a significant translational step toward clinical PBI-CT.

## Materials and methods

### Multimodal validation of lung PBI-CT using an integrated experimental pipeline

To evaluate the future potential of lung PBI-CT for patients, we established an extensive experimental and data analysis pipeline that integrates multiple imaging modalities for direct comparison with clinically established methods. Using a custom-built anthropomorphic chest phantom (ANTHONY, ANThropomorphic pHantom fOr luNg tomographY, MiMEDA GmbH, Schoenau, Germany) [16], six porcine lungs were examined. One representative specimen underwent the full sequential pipeline, including PBI-CT scanning at the SYRMEP (SYnchrotron Radiation for MEdical Physics) beamline at the Italian synchrotron, clinical MSCT, and bronchoscopy at the nearby Hospital of Cattinara, followed by FA vapor fixation and histological analysis. The remaining porcine lungs were primarily used for PBI-CT to expand the dataset and assess technical feasibility. For additional validation of the lung PBI-CT method, two human right lungs, fixed with FA vapor, were imaged in the phantom, obtained from lung transplant recipients.

**Fig. 1:**
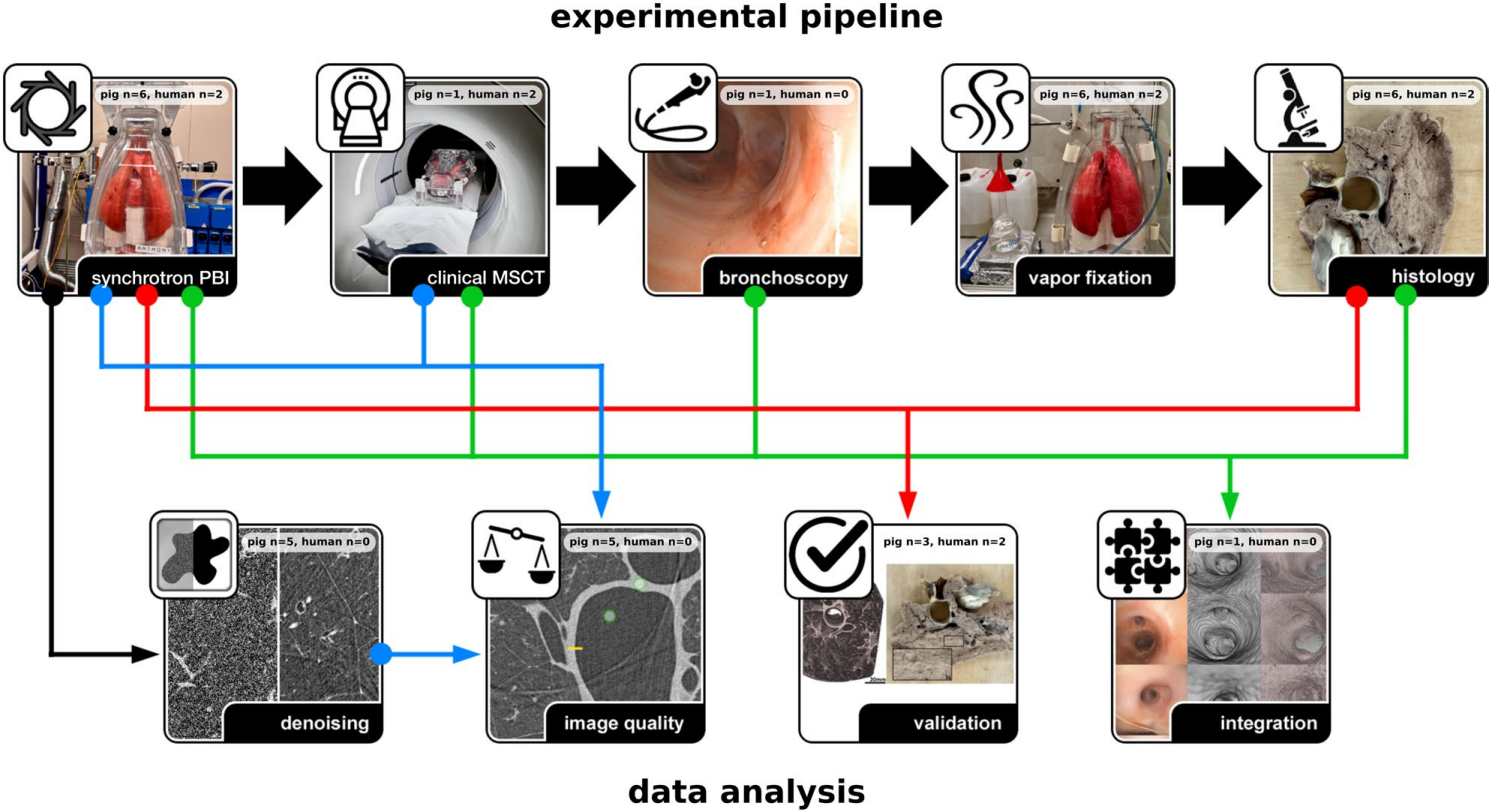
Multimodal experimental and analysis workflow. Lung specimens were imaged within the phantom using synchrotron PBI-CT, clinical MSCT, and bronchoscopy. Afterwards, lungs were FA vapor fixed solely in the inner shell of the phantom and then processed for histology. The PBI-CT data were processed and denoised (black arrow), and image quality was compared to MSCT data (blue arrow) using both quantitative and qualitative measures. The imaging findings in PBI-CT were validated with histology (red arrow). Imaging modalities were integrated within the 3D context of the clinical MSCT data (green arrow). In each panel, the number of pig lungs (out of six) and human lungs (out of two) used for every experimental and analysis step is indicated, providing a clear overview of sample distribution across the study. Human lungs fixed with FA vapor were not imaged with clinical MSCT in the phantom. Instead, the most recent pre-transplant MSCT scans were used as in vivo reference imaging, enabling direct comparison with the ex vivo PBI-CT of the FA vapor-fixed lungs and histopathology. PBI-CT = propagation-based synchrotron phase-contrast computed tomography. MSCT = multislice computed tomography. 3D = three-dimensional. FA = formaldehyde

The acquired data were subjected to four main post-processing and validation strategies:

1. Machine learning-based denoising was used to test whether the radiation exposure levels could be further reduced without compromising the image quality of PBI-CT (Fig. 1, black arrow).
2. The image quality of MSCT was compared to both the regular and denoised PBI-CT data, both quantitatively and qualitatively (Fig. 1, blue arrow).
3. Imaging findings from PBI-CT were directly compared with corresponding histological images, which served as ground truth (Fig. 1, red arrow).
4. Finally, data from all four imaging modalities (PBI-CT, MSCT, bronchoscopy, and histology) were integrated into the three-dimensional (3D) context of the MSCT to evaluate the potential benefits of such a combined analysis (Fig. 1, green arrow).

### Lung specimens and anthropomorphic chest phantom

Six porcine lungs, including trachea, larynx, and parts of the heart, were obtained from mature pigs at a licensed slaughterhouse after veterinary inspection. Residual tissue was removed, and the trachea was severed below the larynx. The lungs were stored and transported to the Italian synchrotron at -20 °C, thawed at room temperature for ≥ 5 h, inspected, and repaired with sutures if necessary. After trimming the trachea, each lung was mounted in the custom-made anthropomorphic chest phantom and secured to the tracheal tube using a cable tie. A thin layer of coconut oil was applied to maintain surface moisture during imaging.

Two human right lungs, one with idiopathic pulmonary fibrosis (IPF) and one with rheumatoid arthritis (RA)-associated usual interstitial pneumonia (UIP) pattern fibrosis, were also examined in the phantom. Both specimens were obtained from the Organ Transplant Center of Hannover Medical School with informed consent and ethics approval (No. 10194 BO K 2022). Handling and preparation of the human specimens are detailed below.

A custom-built anthropomorphic chest phantom ANTHONY (ANThropomorphic pHantom fOr luNg tomographY, MiMEDA GmbH, Schoenau, Germany) [16], developed based on the ArtiCHEST phantom [17], was used for PBI-CT. The phantom consists of two shells: an outer shell mimicking the X-ray attenuation properties of the human chest wall and an inner shell housing the lung. The bone-free version was used, as the photon energy at the SYRMEP beamline was insufficient to penetrate artificial bone structures. The phantom allows secure beamline mounting, vacuum attachment, and optional breathing simulation (not used here). The inner shell incorporates a rigid diaphragm adjustable to lung size and features a silicone seal. It is continuously evacuated using a negative flow pump, ensuring static lung inflation and compensating for potential air leakage. However, this system does not allow for precise measurement of static pressure within the phantom. The portable design enabled transport between the beamline and the nearby Hospital of Cattinara for multislice CT and bronchoscopy. The tracheal adapter allowed external access for bronchoscopy, biopsy, and final FA vapor fixation.

Human lungs were FA vapor-fixed immediately after retrieval to ensure biosafety and stability during transport, following the protocol described in the “Fixation and histology of lung specimens” section.

The use of PBI-CT for FA vapor-fixed lung specimens in the phantom has been previously described by Dullin et al. [16].

### Imaging setup at the Italian synchrotron

**Fig. 2:**
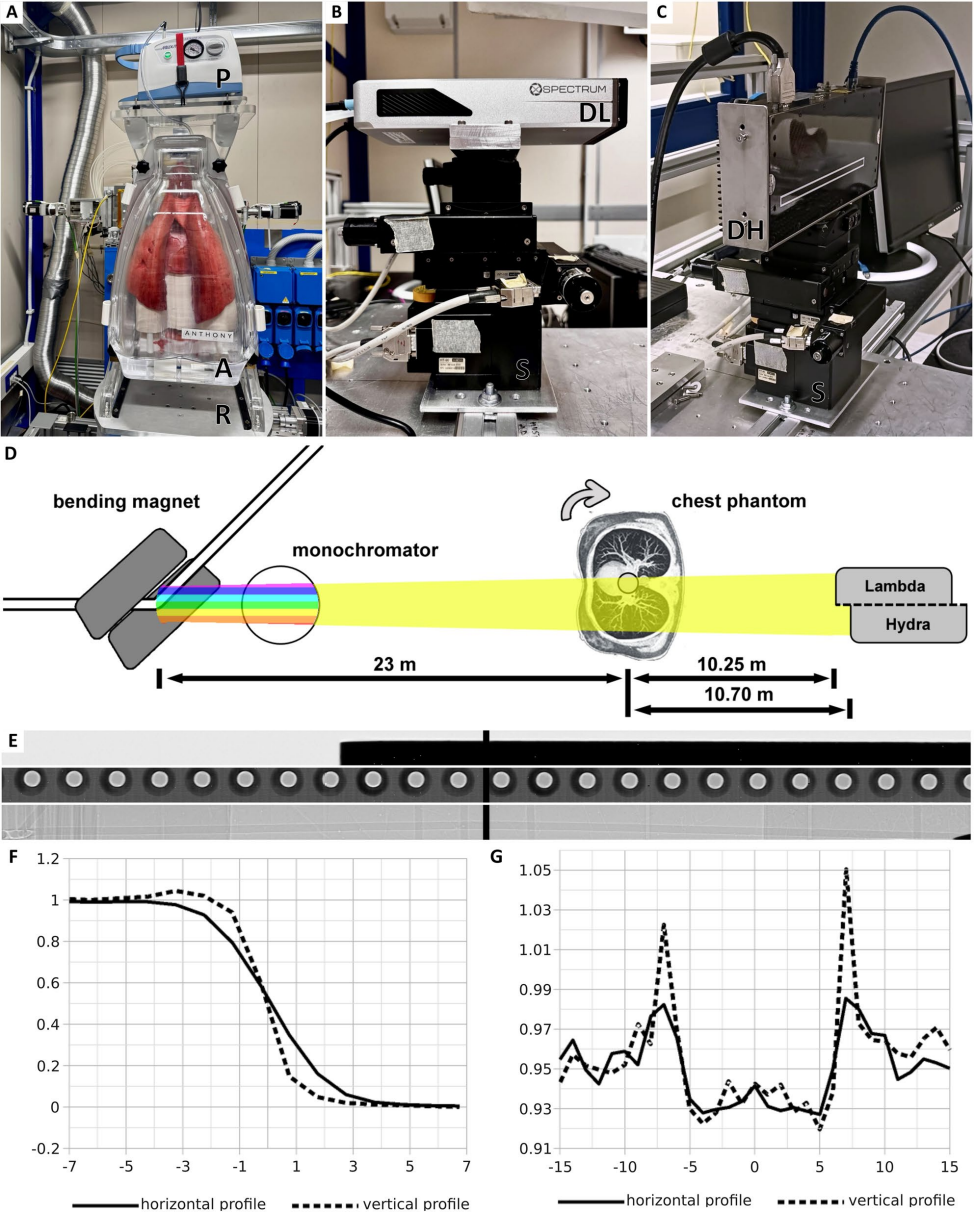
Imaging setup and detector characterization. **A** The phantom (ANTHONY) [A] was equipped with a fresh porcine lung, mounted on top of the rotary unit [R] at the SYRMEP beamline. The vacuum pump [P] was used to keep the lung passively inflated. **B** The LAMBDA [DL] and **C** Hydra photon-counting detector [DH] were each mounted on the same adjustable stage [S]. **D** The X-rays were produced by a bending magnet. A monochromator was used, allowing 40 keV photons to pass through. The phantom was mounted around 23 m away from the source. PBI-CT scans were conducted with the specimen positioned off-center and rotated through a full 360 degrees. LAMBDA was placed 10.25 m and Hydra 10.70 m downstream from the phantom. **E** A copper blade, a circuit board, and polyethylene wires (shown from top to bottom) were used to characterize the setup and the detector. **F** Normalized intensity profiles across the edge of a copper blade, extracted in the horizontal (solid line) and vertical (dashed line) directions, are used for edge analysis. The horizontal profile displays a broader sigmoidal transition region compared to the vertical profile, indicating a source spot blurring of six pixels horizontally and two pixels vertically. **G** Normalized intensity profiles across the surface of polyethylene wires, extracted in the horizontal (solid line) and vertical (dashed line) directions, are used to assess phase contrast-based edge enhancement. Both profiles exhibit periodic intensity peaks at the wire edges, corresponding to phase contrast effects. The edge enhancement reaches *≈* 3% above baseline in the horizontal and up to 8% in the vertical direction. *SYRMEP* SYnchrotron Radiation for MEdical Physics, *PBI-CT * propagation-based synchrotron phase-contrast computed tomography

Ultra-high-resolution imaging was performed at the SYRMEP beamline of the Elettra Synchrotron (Trieste, Italy). The X-ray beam, generated by a bending magnet, exhibited a divergence of 3 mrad. This beam was filtered to an energy of 40 keV with a spectral width of ***≈*** 0.04 keV using a double Si(111) crystal monochromator operating in Bragg configuration [18]. Two different photon counting detectors were used: (i) LAMBDA (X-Spectrum GmbH, Hamburg, Germany) based on Medipix3 technology with a nominal pixel size of 55 μm to image four porcine lungs and the human IPF lung, and (ii) Hydra (Varex Imaging, Salt Lake City, Utah, USA; formerly Direct Conversion AB, Stockholm, Sweden) with a nominal pixel size of 100 μm to image two porcine lungs, and the human RA-associated UIP-pattern fibrosis lung. The beamline was operated using sample-to-detector distances of 10.25 m and 10.70 m, resulting in effective pixel sizes of 38 μm and 67 μm, respectively. 360° off-center scans were performed to increase the lateral field of view (FOV) with 3600 projections. The phantom was positioned around 23 m from the X-ray source. Aluminum filters of up to 7.75 mm thickness were used to realize an entrance radiation exposure level of *≈* 23 mGy. Accounting for the off-center scan, this corresponded to an estimated radiation exposure level of *≈* 11.5 mGy for the exposed tissue, measured by thermoluminescence detectors (TLDs). All relevant acquisition parameters are listed in Table 1. The ANTHONY phantom was mounted on the sample stage for heavy and bulky specimens previously described by Dullin et al. [15]. The imaging setup can be seen in Fig. 2.

**Table 1:** PBI-CT acquisition parameters

Feature	LAMBDA	Hydra
Exposed area [px]	1554 × 65	1914 × 30
Effective pixel size [µm]	38	67
Exposure time [ms]	6.25 (12.50)	10.00
Projections	1800 (3600)	3600
Rotation angle [°]	360	360
Scan time per rotation [s]	11.25 (45.00)	36.00
Avg. entrance radiation exposure [mGy]	5.8 (23.0)*	21.6^**^
Average counts flat	115 (230)	400
Energy threshold [keV]	14	20

Two different settings were used for the LAMBDA detector, with the second setting given in brackets. (*) The calculated relative standard deviation for the TLDs used with the LAMBDA detector (*n* = 5) was 10.4%; (**) for the Hydra detector (*n* = 13), it was 5.5%

*PBI-CT* propagation-based synchrotron phase-contrast computed tomography, *TLDs* thermoluminescence detectors

### PBI-CT image pre-processing and reconstruction

The acquired data were corrected using flat-field images of the incident X-ray beam without the phantom. The used LAMBDA detector consists of four panels from which two were exposed. Each panel is built by several modules, with larger pixels present at the module contact zones. Those larger pixels, along with dead pixels and gaps between the panels, were inpainted using the OpenCV library. In case of the Hydra detector, no additional corrections were needed, as it has a filling rate of 100%.

Then single distance phase retrieval was applied using the TIE Hom algorithm [19], followed by reconstruction with a classical filtered back projection, both implemented in the SYRMEP Tomo Project (STP) software [20]. In addition to this standard reconstruction pipeline, AI-based denoising was performed in sinogram space prior to inpainting and phase retrieval. To achieve this, end pairs of sinograms were generated by using every second line of the original sinogram. A ResNet architecture was used as described in the Noise2Noise strategy by Lehtinen et al. [21]. A loss of function mean square error was applied. All parameters of the pre-processing and reconstruction process are listed in Table 2. Training was performed separately for the LAMBDA and Hydra data on a computer with the following specs: CPU = AMD Ryzen Threadripper PRO 5965WX, RAM = 192 GB, and GPU = NVIDIA ATX 6000 (48 GB).

**Table 2:** Summary of the PBI-CT pre-processing and reconstruction parameters

Feature	LAMBDA	LAMBDA denoised	Hydra	Hydra denoised
Projections	1804	1804	1800	1800
Effective entrance dose [mGy]^§^	11.5^*^	11.5^*^	10.8^**^	10.8^**^
Delta-to-beta ratio	2000	2000	2000	2000
Inpainting	Yes	Yes	No	No
Denoising	No	Yes	No	Yes

(§) The effective entrance radiation exposure considers that the scan was performed in 360° off-center mode, meaning that most of the tissue was exposed for only half of the total scanning time. (*) The calculated relative standard deviation for the TLDs used with the LAMBDA detector (*n* = 5) was 10.4%; (**) for the Hydra detector (*n* = 13), it was 5.5%. PBI-CT = propagation-based synchrotron phase-contrast computed tomography.  *TLDs* thermoluminescence detectors

### Clinical imaging with MSCT and bronchoscopy

**Fig. 3:**
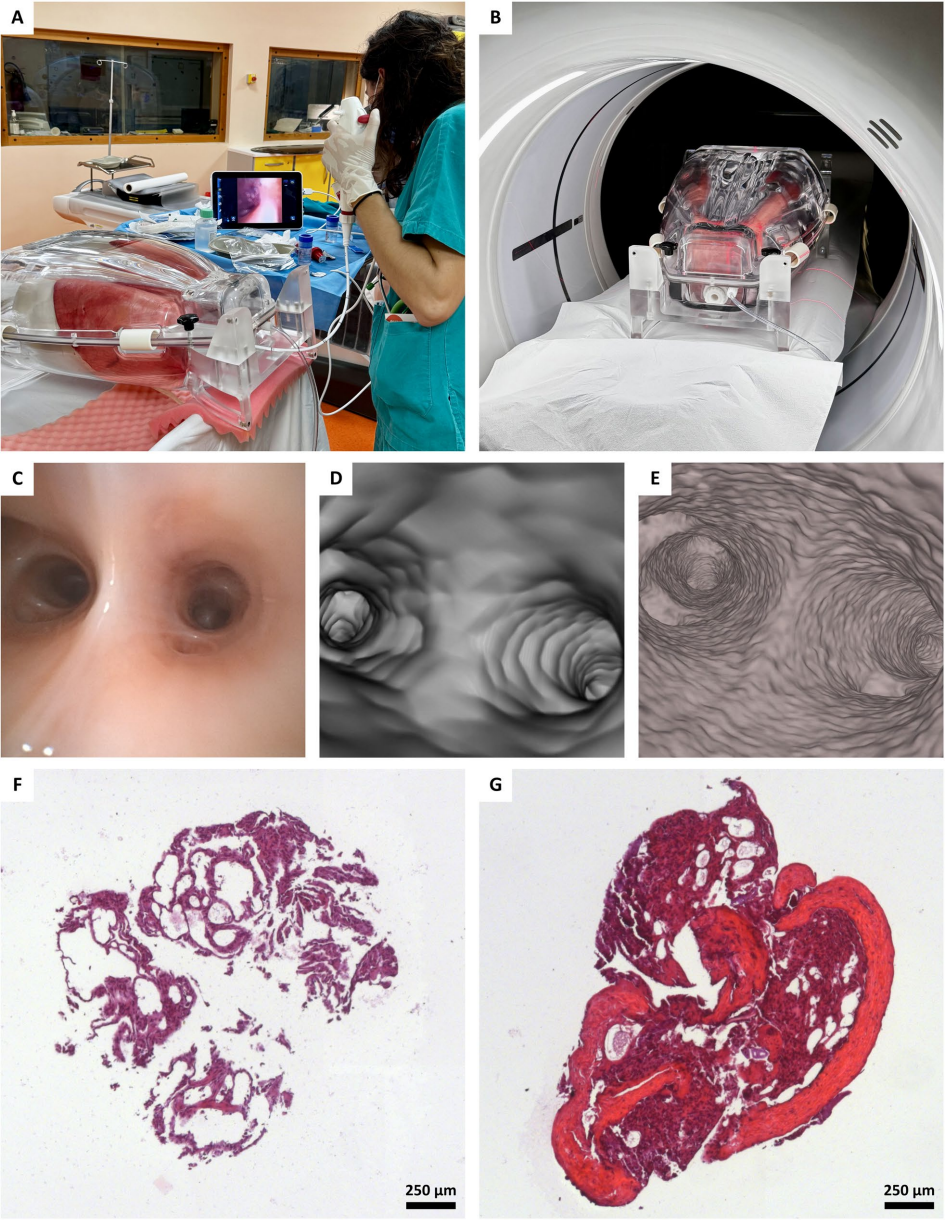
Sequential imaging with bronchoscopy, MSCT, and PBI-CT. **A** Bronchoscopy with biopsy sampling was performed on a passively inflated porcine lung within the phantom. **B** The same lung specimen is shown during MSCT acquisition. **C** Still frame of the obtained bronchoscopy video, **D** the 3D volume-rendered MSCT dataset, and **E** the 3D volume-rendered PBI-CT dataset (LAMBDA), all shown in the same view. The PBI-CT dataset reveals a markedly higher level of structural detail, including the architecture of the airway wall, and superior resolution of smaller, more peripheral airways compared to bronchoscopy and MSCT (Video, Supplementary Material 1). **F** and **G** show two H&E-stained slices from biopsies obtained during the bronchoscopy of the porcine lung, highlighting the alterations in tissue quality and revealing strongly compromised structural integrity of the specimens, which are typical artifacts introduced by this procedure. *MSCT* multislice computed tomography, *PBI-CT* propagation-based synchrotron phase-contrast computed tomography, *3D* three-dimensional. *H&E* hematoxylin and eosin

Clinical MSCT and bronchoscopy were conducted after PBI-CT on one of the six porcine lungs at the Hospital of Cattinara, Italy (Fig. 3).

MSCT was acquired using a Revolution™ Frontier CT (GE Medical Systems, Chicago, Illinois, USA) in helical scan mode with 120 kVp, 146 mA, 73.8 mAs, 0.625 mm slice thickness, and 0.76 mm pixel spacing. The scan covered 28.8 cm in 1.80 s. Reconstruction was performed with the proprietary software of the scanner. Using the same type of TLDs as in the PBI-CT scan, the entrance radiation exposure was measured to be *≈* 15.0 mGy.

Subsequent bronchoscopy was performed by a pneumologist using a single-use flexible bronchoscope Ambu^®^ aScope™5 Broncho HD 5.6/2.8 (Ambu A/S, Ballerup, Denmark).

For the two human right lungs obtained from transplant recipients, one with IPF and one with RA-associated UIP-pattern fibrosis, pre-transplant MSCT scans were retrieved from the patients’ medical records for analysis. The IPF scan was acquired using a Toshiba Aquilion PRIME scanner (Canon Medical Systems, Otawara, Tochigi, Japan), and the RA-associated UIP-pattern fibrosis scan was acquired using a Siemens Somatom X.ceed scanner (Siemens Healthineers, Erlangen, Germany). Acquisition parameters were as follows: helical mode, 120 kVp, 50 mAs, 0.68 mm pixel spacing, and 1.0 mm slice thickness for the IPF patient; and helical mode, 100 kVp, 337 mAs, 0.98 mm pixel spacing, and 1.0 mm slice thickness for the RA-associated UIP-pattern fibrosis patient. Reconstruction was performed using standard lung and soft-tissue kernels according to the clinical protocol at Hannover Medical School. These scans provided in vivo reference imaging of the lungs prior to transplantation, allowing direct comparison with subsequent ex vivo PBI-CT and histopathology.

### Quantitative image comparison

For quantitative image comparison we used established metrics, including the contrast-to-noise ratio (CNR), the Full Width at Half Maximum (FWHM) of an edge profile, and the radially averaged power spectra. The following equation was used to calculate CNR:

$$CNR=\frac{|{\mathrm{S}}_{1}-{\mathrm{S}}_{2}|}{\sqrt{0.5\mathrm{*}({\sigma\:}_{1}^{2}+{\sigma\:}_{2}^{2}\:)}}$$

where *S*_*i*_ and *σ*_*i*_ represent the mean gray value and standard deviation, respectively, within two adjacent regions (1 and 2) containing homogeneous tissues. Three measurements were performed for each reconstructed data set at approximately the same anatomical regions.

Image sharpness was assessed by analyzing edge profiles within larger bronchial passages, specifically from the air-filled lumen to the bronchial wall, using three slices from each reconstructed data set. The absolute values of the first derivative were approximated via the difference coefficient and normalized to a range of 0 to 1. The FWHM was then determined with interpolated subpixel precision and expressed in terms of the effective pixel size for each detector.

To compute the radially averaged power spectra, regions of equivalent size (in mm^2^) and containing comparable anatomical structures were selected across all five data sets: MSCT, LAMBDA, LAMBDA denoised, Hydra, and Hydra denoised. Maintaining an unbiased perspective pertaining to the differences in the overall brightness of the data, the power at zero frequency was set to zero, and the remaining power was radially averaged and normalized, yielding a total power of 1.

### Qualitative image comparison

Image quality was assessed qualitatively by radiological scoring. To ensure blinding to imaging modality, two-dimensional (2D) circular regions (48 mm radius), matching the FOV of the PBI-CT scans, were extracted from all the PBI-CT (LAMBDA, LAMBDA denoised, Hydra, and Hydra denoised) and clinical MSCT data sets. These regions were manually selected to depict comparable lung areas. For uniform visual inspection, all images were scaled without interpolation to the same matrix size of 2500 × 2500 px^2^ and displayed with equalized brightness and contrast. A custom Python script randomly paired images (*n* = 50), designating one as the reference and the other as the comparison.

Six independent medical experts (five radiologists, one pneumologist) assessed 14 image features (overall impression, spatial resolution, contrast resolution, image noise, artifacts, bronchi, bronchioles, smallest visible airway, extra-/intralobular veins/arteries, and inter-/intralobular septa) using a 5-point scale ranging from − 2 to + 2 (-2: much worse, -1: slightly worse, 0: comparable, + 1: slightly better, and + 2: much better than the reference image).

### Fixation and histology of lung specimens

After imaging, lungs were kept inflated within the phantom’s inner shell using a negative pressure pump and fixed with FA vapor, as described by Dullin et al. [16]. Fixation was continued for *≈* 6 h, until the lung surface had completely changed from a pinkish to a brownish color, with only minimal shrinkage observed by gradually reducing the suction rate of the pressure pump. Fixed lungs were stored for roughly four weeks in a sealed plastic container with a small amount of FA at the bottom, to maintain a moist environment.

Lamellae (***≈*** 1 cm thick) were cut in an area intersecting the FOV of the PBI-CT scans. Photos of these lamellae were taken to facilitate correlation with PBI-CT and, when available, MSCT. Lamellae were sectioned into pieces (***≈*** 2 × 3 cm^2^), chemically dehydrated by an ascending ethanol series, and embedded in paraffin. Tissue blocks were processed following the standard workflow for formalin-fixed and paraffin-embedded (FFPE) samples, sectioned (2 μm), and stained with hematoxylin and eosin (H&E) or Masson-Goldner using standardized protocols. Images were acquired at 5x magnification, and selected regions at 10x and 20x magnification, using an inverted microscope (Axiovert 200 Zeiss, Oberkochen, Germany).

FA vapor fixation preserved the lung’s physiological shape, however, the specimens remained partially flexible, with observed shrinkage by roughly 10 to 20% in linear dimension.

### Software

Image pre-processing was performed with custom Python scripts using OpenCV (opencv.org) and TensorFlow (www.tensorflow.org). For PBI-CT data sets, phase retrieval and reconstruction were accomplished using the STP software (Version 1.6.3) [19, 20], while the stitching of individual scans was handled within Python. Fiji (NIH) was employed for basic image handling and measuring the CNR and FWHM [22]. Custom Python scripts generated radial power spectra and image pairs for visual scoring. 3D volume rendering, airway segmentation, and correlation of PBI-CT, MSCT, bronchoscopy, and histology data were performed using VGStudioMax Version 3.1 and 3.4.5 (Volume Graphics GmbH, Heidelberg, Germany). Video editing utilized iMovie Version 10.4.2 (Apple Inc., Cupertino, California, USA) and DaVinci Resolve Version 20.0 (Blackmagic Design Pty. Ltd., Port Melbourne, Australia). Statistical analyses were performed using the Python packages seaborn (seaborn.pydata.org) and statannotation (pypi.org/project/statannotations). Figures were compiled using OpenOffice (Apache Software Foundation), Photoshop 6.0 (Adobe Inc., San Jose, California, USA), and Inkscape (Inkscape Project).

## Results

### Characterization of the LAMBDA detector demonstrated feasibility for PBI-CT imaging of human-sized lungs

For the first time, the photon-counting detector LAMBDA (55 × 55 µm^2^ nominal pixel size) was utilized and characterized for PBI-CT imaging of human-sized lungs within the ANTHONY phantom. Its performance was compared to that of the previously used and characterized single photon-counting detector Hydra (100 × 100 µm^2^ nominal pixel size) [15, 16, 18]. Figure 2A shows the ANTHONY phantom (A) mounted on top of the rotary unit (R) within the experimental hutch of the SYRMEP beamline. A negative pressure pump (P) was positioned above the phantom to maintain passive lung inflation. Figure 2B illustrates the LAMBDA detector (DL), and Fig. 2C, the Hydra detector (DH), each on the same stage (S) that allows tilting for precise alignment. Figure 2D provides an overview of the experimental setup. This configuration resulted in an effective pixel size of 38 μm and 67 μm, respectively (Table 1). Additionally, under these conditions, the projected source spot size was expected to be reduced to approximately half the actual source spot size. Detector characterization employed a 5 mm-thick copper sheet, a circuit board, and crossed polyethylene wires (Fig. 2E). From the copper sheet, horizontal and vertical edge profiles were measured (Fig. 2F). In the horizontal direction, the edge was blurred across approximately six pixels, corresponding to a blurriness of 230 μm, while in the vertical direction, the blurriness was 77 μm over two pixels. Based on the 2.24 ratio between source-to-sample and sample-to-detector distance, the estimated source spot size was 515 μm horizontally and 172 μm vertically. The circuit board, with its known spacing between soldering points, was used to validate the effective pixel size. Differences in the horizontal and vertical extents of the source spot were also reflected in the corresponding line profiles of the projection data for the 0.5 mm polyethylene wires (Fig. 2G). Edge enhancement was observed in both directions, confirming sufficient spatial coherence and an appropriate sample-to-detector distance for effective PBI-CT. However, the edge enhancement was notably higher in the vertical direction (8%) than in the horizontal direction (3%). The results of the characterization suggested that the LAMBDA detector was well-suited for PBI-CT imaging in this setup. However, the source spot size introduced blurring, that prevented achieving spatial resolutions near the effective pixel size of 38 μm.

### PBI-CT outperformed MSCT and bronchoscopy in visualizing peripheral lung structures in a porcine model

To assess the diagnostic potential of PBI-CT relative to clinical standards, one representative porcine lung was sequentially imaged using PBI-CT, MSCT, and bronchoscopy within the transportable ANTHONY phantom (Fig. 3A, B). Acquisition parameters for PBI-CT (effective pixel size 0.038 mm) are detailed in Table 1, and for MSCT (pixel spacing 0.76 mm) in the Methods section. Both modalities were performed with comparable entrance radiation exposures, as measured using TLDs; MSCT: ≈15.0 mGy, PBI-CT: ≈23.0 mGy (estimated tissue dose: ≈11.5 mGy). For PBI-CT, the initial entrance radiation dose was ≈ 23.0 mGy. However, due to the off-center scan geometry, the dose to the actual exposed tissue within the FOV was ≈ 11.5 mGy. Acquisition times were 1.8 s for 28.8 cm for MSCT (helical mode) and 11.25–36.0 s per rotation for PBI-CT (LAMBDA/Hydra detectors) in step-and-shoot mode with a vertical FOV of *≈* 2.0–2.5 mm. Multiple scans were performed along the vertical axis for both photon-counting detectors, and the resulting data sets were stitched together vertically to achieve an extended anatomical coverage.

Sequential imaging with bronchoscopy, MSCT, and PBI-CT emphasized the superior resolving power of PBI-CT (Fig. 3C-E). Whereas bronchoscopy was limited to proximal bronchi and MSCT depicted the overall bronchial architecture, PBI-CT provided distinctly higher soft-tissue contrast and visualized peripheral airways that were not discernible with either reference modality (Video, Supplementary Material 1). Histologic assessment of bronchoscopic biopsy samples confirmed procedure-related artifacts and loss of structural integrity, underscoring the invasiveness and diagnostic limitations of conventional sampling (Fig. 3F, G).

**Fig. 4:**
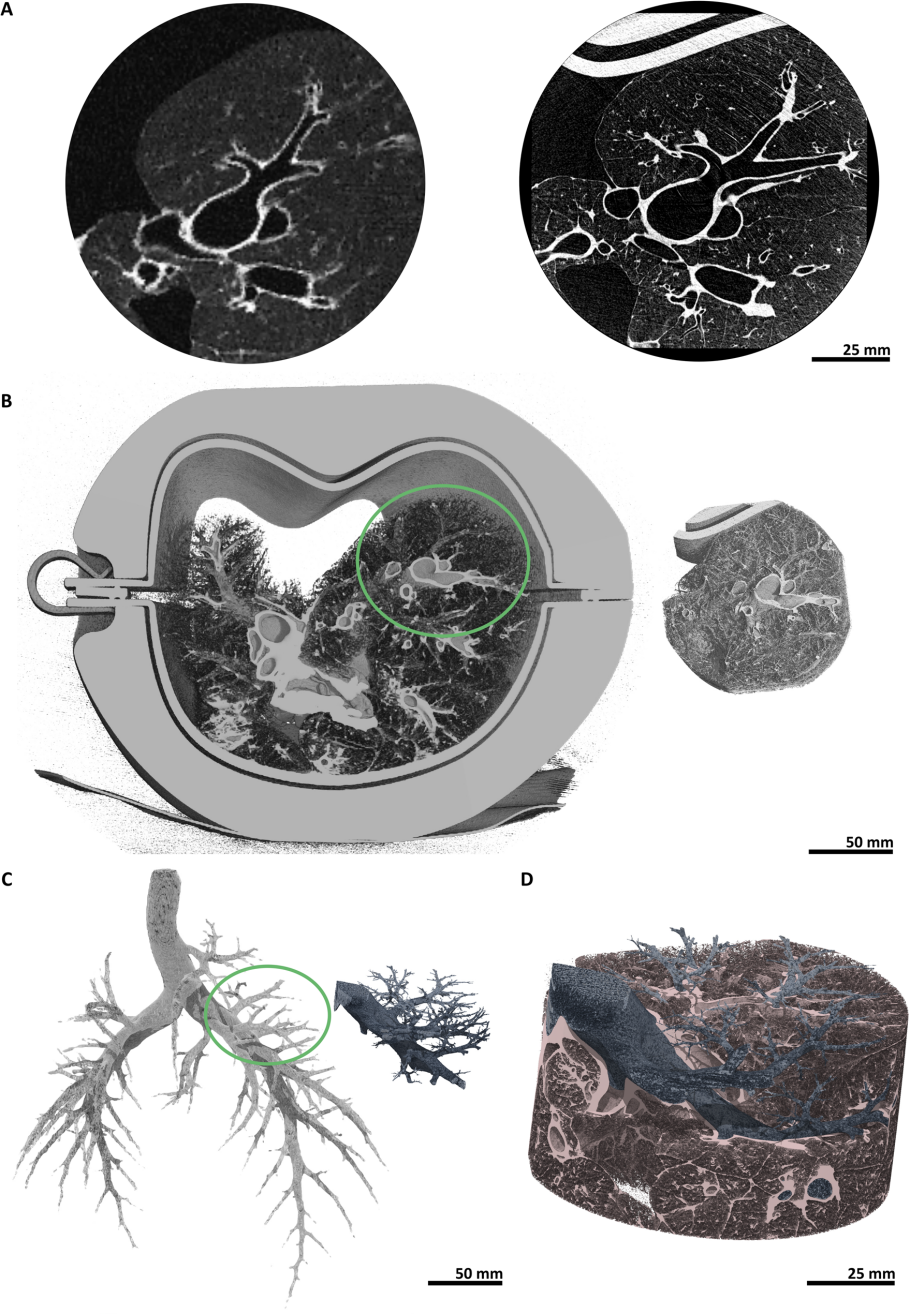
Registration and segmentation: MSCT vs. PBI-CT. **A** 2D axial slices of a pig lung within the phantom acquired using clinical MSCT (left) and PBI-CT with the LAMBDA detector (right) are shown within manually selected circular regions to match the field of view of the PBI-CT scan. **B** 3D volume renderings of the entire phantom acquired using clinical MSCT (left) and the corresponding local region imaged with PBI-CT using the LAMBDA detector (right). The green circle on the MSCT image marks the region corresponding to the PBI-CT view. **C** To demonstrate the gain in diagnostic power, the bronchial tree was segmented in both the MSCT data set (grey, left) and the PBI-CT data set (blue, right). The green circle on the MSCT image marks the region corresponding to the PBI-CT view. In the segmented PBI-CT data, at least three additional airway generations can be observed. **D** The segmented airways from the PBI-CT dataset, shown in panel **C**, are embedded within the 3D-rendered PBI-CT volume to emphasize fine parenchymal structures, like intralobular septa, clearly visible in the high-resolution PBI-CT images. *MSCT* multislice computed tomography, *PBI-CT* propagation-based synchrotron phase-contrast computed tomography, *2D* two-dimensional, *3D* three-dimensional

Figure 4A shows 2D axial slices from MSCT (left) and PBI-CT (right, LAMBDA detector). Image brightness and contrast were harmonized for optimal comparison, with a cropped, circular region reflecting the smaller PBI-CT FOV. The limited FOV is due, partly, to the narrower LAMBDA detector compared to the available synchrotron beam at SYRMEP, and partly, to the current beam itself being too narrow to cover the full width of a human-sized lung. The planned SYRMEP-LS upgrade will provide a beam large enough for full, cross-sectional lung imaging when paired with wider detectors. PBI-CT demonstrates clearly superior spatial resolution, visualizing peripheral airways and vessels, well beyond MSCT. Figure 4B shows 3D volume renderings, allowing direct anatomical comparison, despite slight mismatch introduced by varying phantom orientation (MSCT: horizontal; PBI-CT: vertical) and expansion state after lung reinflation between modalities, necessary due to the cable-operated vacuum pump used. In Fig. 4C, direct comparison of segmented bronchial trees reveals PBI-CT resolving at least three additional airway generations versus MSCT according to Bauer et al.’s classification [23]. Figure 2D overlays segmented bronchi on a PBI-CT volume rendering, visualizing delicate structures such as interlobular septa.

### Trade-off between contrast and sharpness in PBI-CT

**Fig. 5:**
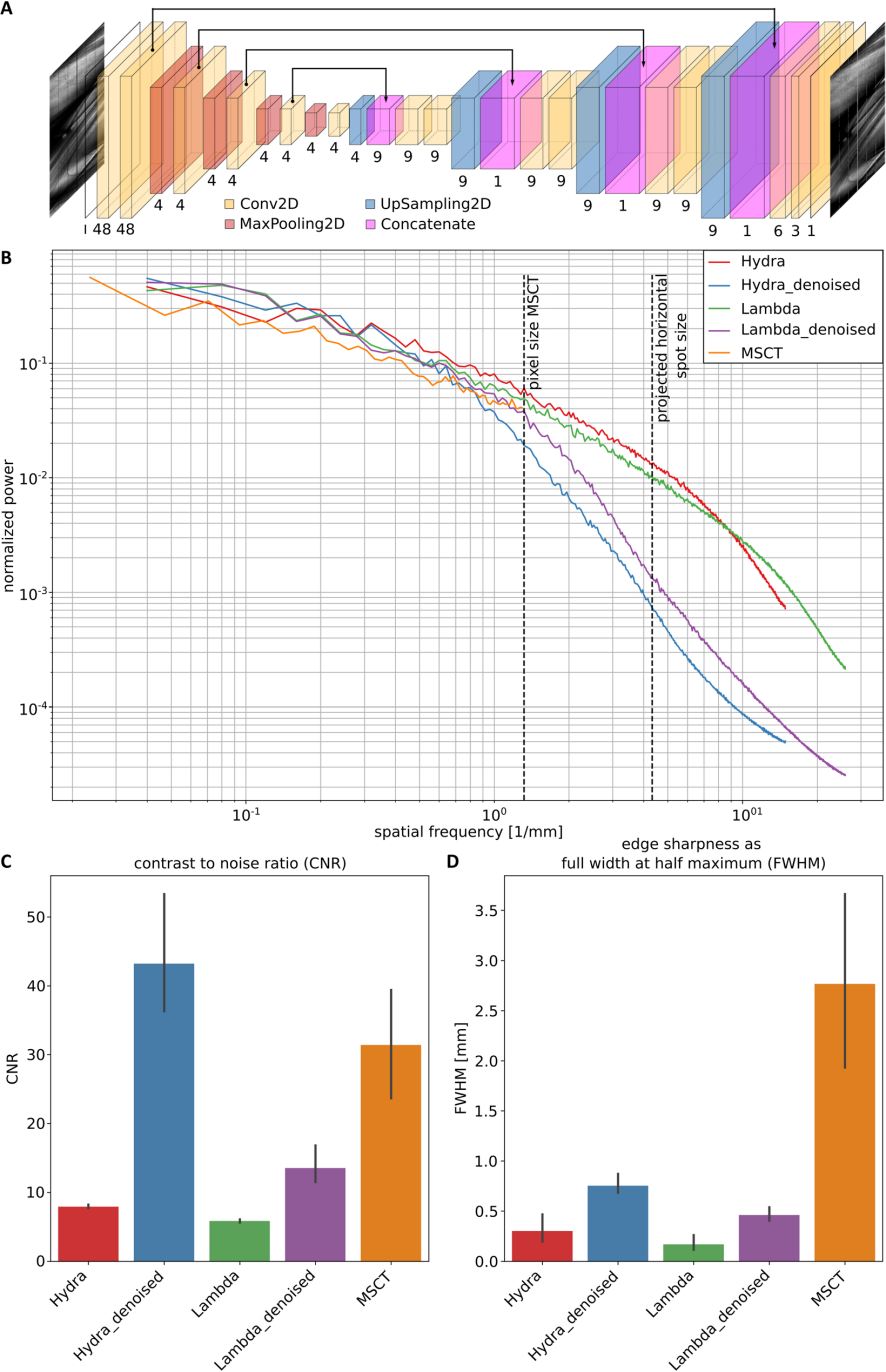
Quantitative image quality assessment. **A** Scheme of the ResNet architecture used for denoising, including Conv2D, MaxPooling2D, UpSampling2D, and Concatenate layers, shown by different colors. **B** Radial averaged power spectra, showing similar power for all five data sets (Hydra, Hydra denoised, LAMBDA, LAMBDA denoised, and MSCT) until the cut-off frequency of MSCT. At higher spatial frequencies, Hydra shows slightly better performance than LAMBDA. Especially in the very high frequencies, a strong noise reduction by the denoising can be seen. **C** CNR measurements confirm the data of the power spectra, showing a higher CNR for Hydra compared to LAMBDA data, and a strong increase of CNR in the denoised data, respectively. MSCT shows the highest CNR in comparison to the non-denoised data. **D** The edge sharpness, measured in mm of FWHM, shows the smallest value for LAMBDA, followed by Hydra data. The denoised data clearly has a decreased edge sharpness. However, this effect is less pronounced than the increase in CNR. All PBI-CT data sets (Hydra, Hydra denoised, LAMBDA, LAMBDA denoised) surpass the edge sharpness of MSCT. *MSCT* multislice computed tomography *CNR* Contrast-to-noise ratio, *FWHM* full width at half maximum, *PBI-CT* propagation-based synchrotron phase-contrast computed tomography

We investigated whether radiation exposure could be further reduced without compromising image quality. To this end, we applied machine learning-based denoising to the PBI-CT data. Both LAMBDA and Hydra datasets underwent AI-based denoising using the Noise2Noise [21] approach, where a ResNet model (Table 3) was trained on paired sinograms from alternating projections (Fig. 5A). To ensure comparability between denoised and original datasets under equivalent radiation dose conditions, reconstructions of the non-denoised data were performed using only 50% of the available projections. The estimated tissue dose for the full set of projections was calculated as 11.5 mGy, which is reduced to 5.75 mGy when using only half of the projections.

**Table 3:** AI-based denoising parameters

Feature	LAMBDA denoised	HYDRA denoised
Network architecture	4-Level ResNet	4-Level ResNet
Loss function	Mean square error	Mean square error
Image size	1554 × 1900	1915 × 1800
Image data	4368 pairs	4520 pairs
Train / test split	80/20	80/20
Specimens included	3 porcine lungs	4 porcine lungs
Scans included	125	269
Epochs	10^*^	30

*Denotes that the training was interrupted, as the early stopping conditions were met

*AI* artificial intelligence

Power spectra (Fig. 5B) were similar across all datasets at low frequencies. At higher frequencies, non-denoised datasets retained more power, especially for Hydra, which has larger pixels (100 × 100 μm² vs. LAMBDA’s 55 × 55 μm²). Denoising reduced high and mid-frequency power, indicating a low-pass filtering effect. This was reflected in the CNR gains, 2.6 (Hydra) and 2.3 (LAMBDA), but also in broader edge profiles, as the FWHM increased (Hydra = 2.5, LAMBDA = 2.8) (Fig. 5C, D).

Artifacts were more evident in LAMBDA data, likely from synchronization issues between frame rate and rotation. Low photon counts (mean 4.7 photons per projection) under 40 keV beam energy may have amplified differences between denoised and raw images.

### Radiological scoring highlighted superior image quality of PBI-CT compared to MSCT

Six expert readers (five radiologists and one pneumologist) assessed 50 randomly generated image pairs from five datasets (MSCT, LAMBDA, LAMBDA denoised, Hydra, and Hydra denoised). They scored 14 image quality features using a 5-point scale from − 2 to + 2, where − 2 indicated the feature in the comparison image was “much worse”, -1 “slightly worse”, 0 “comparable”, + 1 “slightly better”, and + 2 “much better” than in the reference image.

**Fig. 6:**
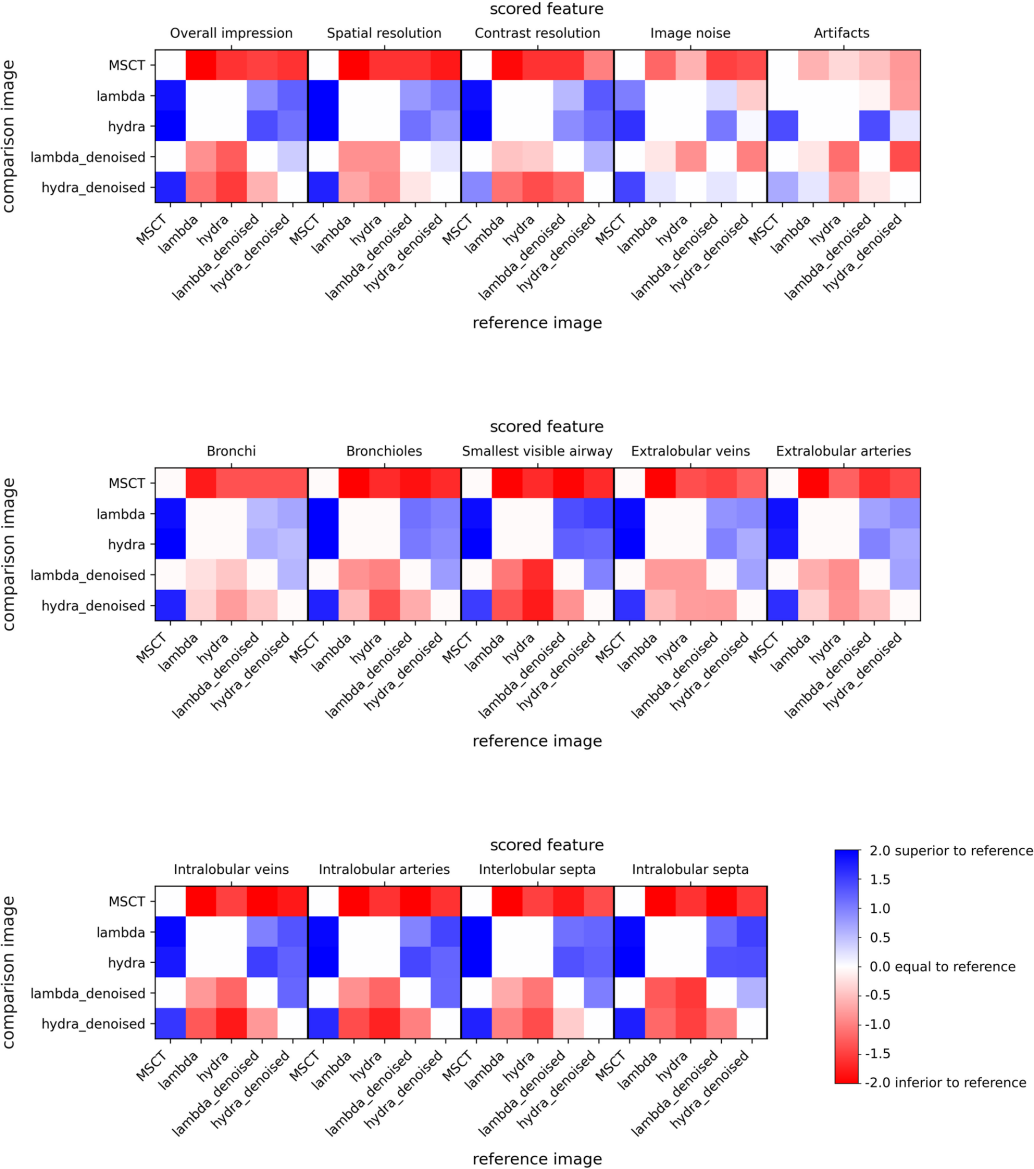
Radiological scoring across modalities. Randomly generated pairs of images were scored by six independent readers for a set of 14 features spanning general quantities such as “overall impression” to the visibility of specific anatomical structures like “intralobular septa”. Scores ranged from − 2 to + 2, where − 2 indicated the feature in the comparison image was “much worse”, -1 “slightly worse”, 0 “comparable”, + 1 “slightly better”, and + 2 “much better” than in the reference image. Image data sets from MSCT and PBI-CT (LAMBDA, Hydra, LAMBDA denoised, and Hydra denoised) were compared. Clearly, LAMBDA and Hydra data outperform MSCT data across all features by two scores (blue), except for image noise and artifacts presence. Hydra denoised data is found to be scored higher quality than MSCT data, while LAMBDA denoised data is scored equally to MSCT data (white). In direct comparison, the original LAMBDA and Hydra data is rated better than their respective denoised versions. MSCT = multislice computed tomography. PBI-CT = propagation-based synchrotron phase-contrast computed tomography

In Fig. 6 the heat maps show that PBI-CT datasets outperformed MSCT in nearly all categories. LAMBDA and Hydra scored equally overall. However, for artifacts and noise, Hydra outperformed MSCT more consistently, while LAMBDA was rated similarly.

Denoised LAMBDA images were rated worse than non-denoised for noise and artifacts, despite quantitative CNR gains. Readers appeared to value sharpness over noise suppression, consistent with the observed low-pass effects.

The scoring confirmed PBI-CT’s diagnostic potential, though it also showed that the projected source spot size (≈ 200 μm) limited resolution, despite small pixel sizes of both detectors.

### Hierarchical validation of lung PBI-CT achieved via in-phantom FA vapor fixation

Validation against histology was essential due to PBI-CT’s unprecedented resolution. To maintain anatomical integrity, lungs were fixed with FA vapor while inflated in the phantom. This preserved their geometry, allowing correlation of histology, serving as ground-truth, with PBI-CT images.

**Fig. 7:**
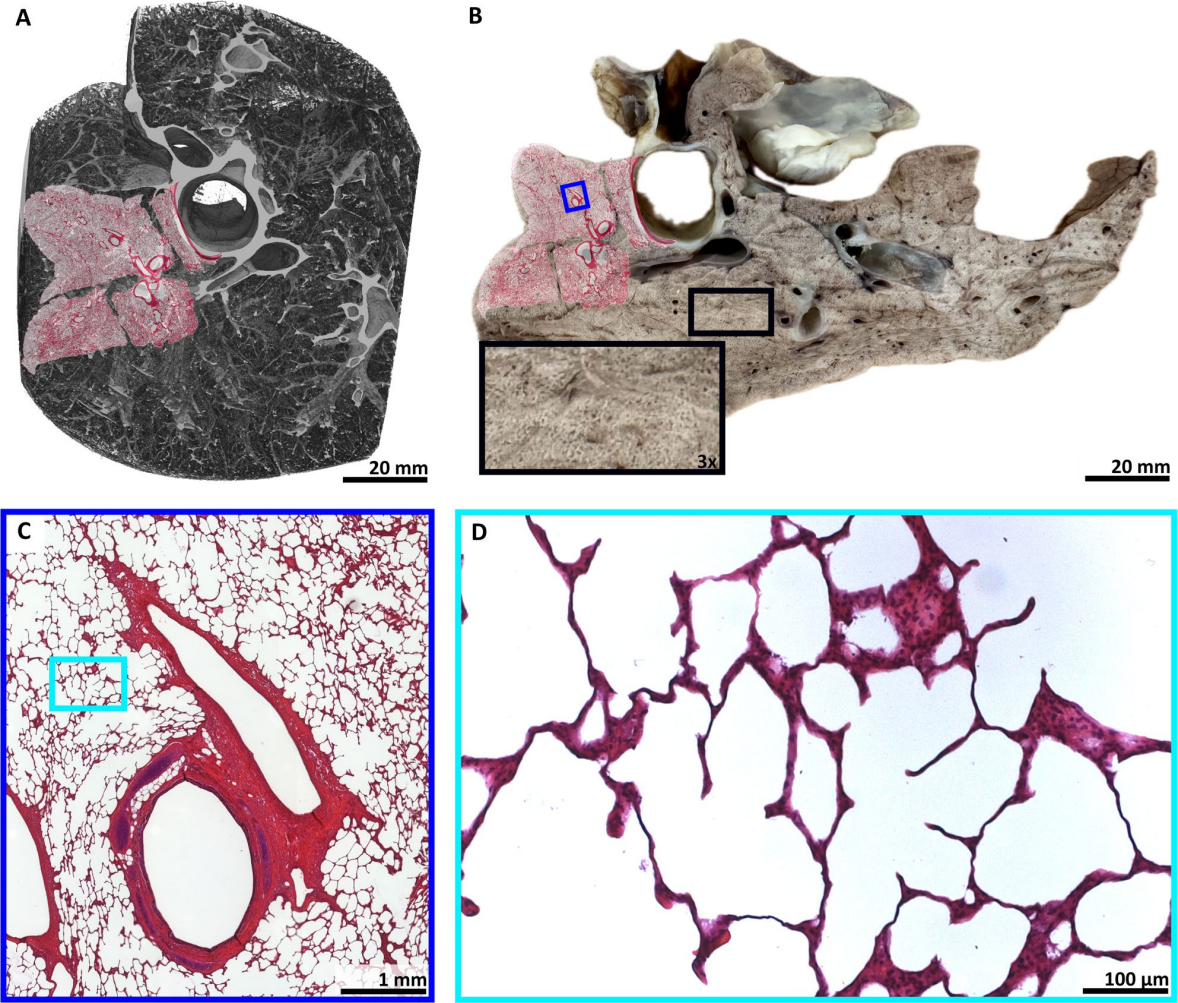
Histological validation of PBI-CT findings. **A** 3D volume rendering of a local area of a PBI-CT scan of a porcine lung, obtained with the LAMBDA detector, in which multiple scans were vertically stitched, and volume virtually cut. The subsequently performed H&E-stained histology is shown as an overlay. **B** A photo taken of a lamella from the same FA vapor fixed lung shown in **A**, demonstrating its macroscopic appearance. While clearly the same anatomical region is shown, the fixed specimen appears slightly deformed compared to **A**. The detail view (black rectangle) indicates well preserved lung tissue. Subsequently performed H&E-stained histology is shown as an overlay. **C** Detail view of the histological data indicated by the blue rectangle in **B**. **D** Further magnification of the light microscopic data indicated by the turquoise rectangle in **C** reveals thin, straight, and non-swollen appearing interalveolar septa, indicating good tissue quality with a physiological alveolar expansion state, preserved by the FA vapor fixation technique, which maintained the air-filled condition. The demonstrated data shows that lung structures imaged by PBI-CT can successfully be validated by histology after using FA vapor fixation. *3D* three-dimensional, *PBI-CT* propagation-based synchrotron phase-contrast computed tomography, *H&E* hematoxylin and eosin, *FA* formaldehyde

Figures 7A, B show H&E-stained slices overlaid on PBI-CT renderings and macroscopic lung images. The tissue remained well-preserved and comparable in morphology. Histological sections (Fig. 7C, D) demonstrated preserved acinar structure and septae, indicating that alveolar inflation was maintained during fixation.

This hierarchical approach, from PBI-CT to FFPE-based histology, verified the fidelity of PBI-CT imaging.

### PBI-CT enabled detection and differentiation of subtle lung pathologies beyond MSCT capabilities

**Fig. 8:**
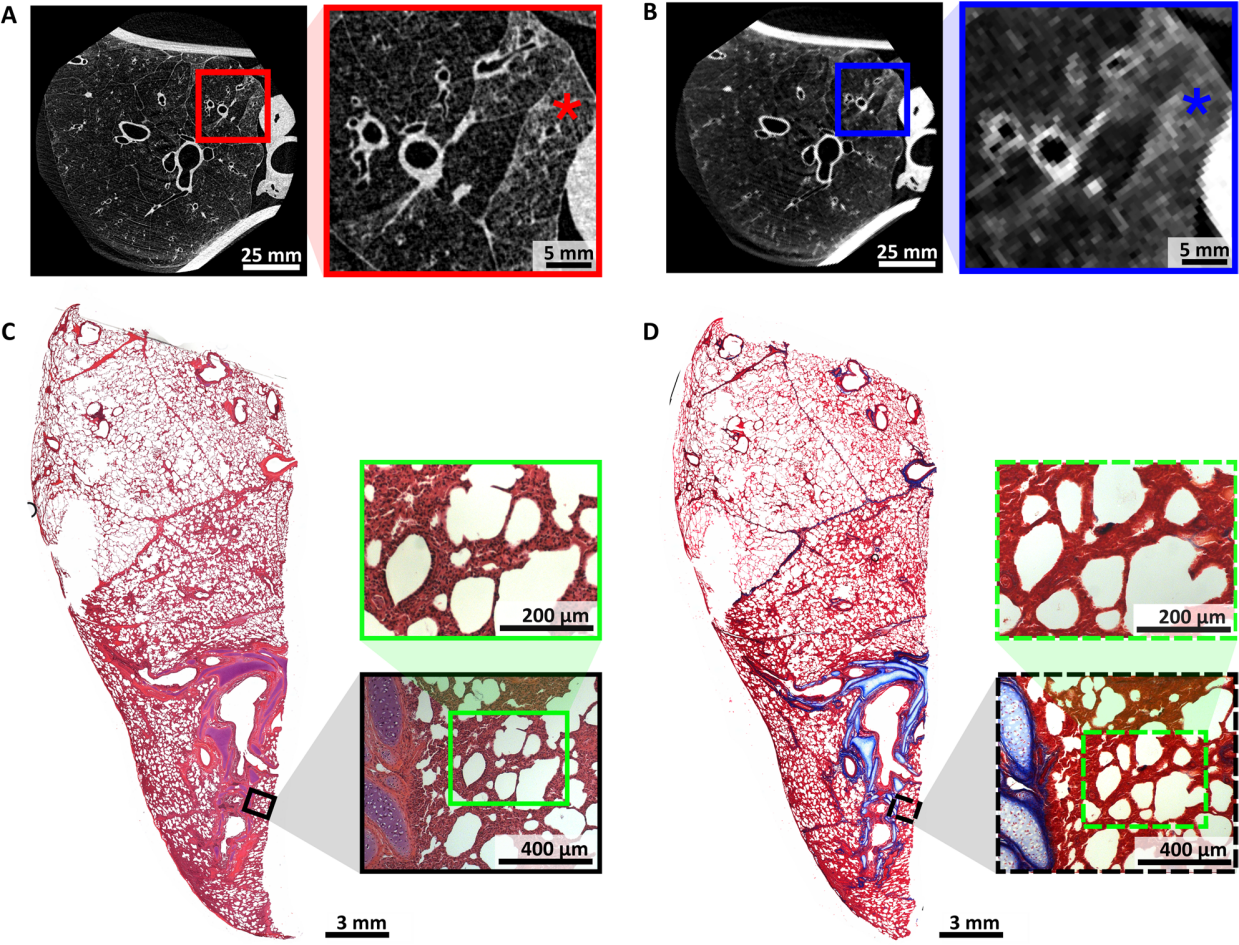
Incidental detection of pathological features in a porcine lung. **A** A slice from a PBI-CT scan (LAMBDA) of a porcine lung. The detail view (red rectangle) shows that several lobules appear to be denser (*). **B** The same slice after spatial down sampling and adjusted in slice thickness, to simulate MSCT conditions. At this reduced resolution, as highlighted in the detail view (blue rectangle), the dense-appearing individual secondary pulmonary lobules (*) can no longer be clearly distinguished. **C** An H&E-stained histological section from a FFPE tissue block of approximately the same lung region reveals a severe loss of acinar microarchitecture with thickening of the interalveolar septa, as seen in the higher magnification insets (black and green rectangles). **D** The corresponding Masson-Goldner-stained section provides further insight into the underlying pathology. A typical distribution of collagen is visible within the interlobular septa, which clearly demarcate the pulmonary lobules. Collagen is also present within the bronchopulmonary bundles, where it forms the characteristic peribronchovascular connective tissue sheath. In contrast, only fine collagen fibers are detected within the parenchyma, including the seemingly thickened septa, as seen in the higher magnification insets (black and green dashed rectangles). These findings suggest that the alveoli in this region have collapsed, likely due to microatelectasis, thereby mimicking septal thickening. *PBI-CT* propagation-based synchrotron phase-contrast computed tomography *MSCT* multislice computed tomography *H&E* hematoxylin and eosin, *FFPE* formalin-fixed and paraffin-embedded

Although the porcine specimens were clinically assessed as healthy, PBI-CT unexpectedly revealed “ground-glass opacities” in several lobules of one specimen, identified incidentally (Fig. 8A). High-resolution imaging revealed retained, air-filled structures within these opacities, indicating a non-homogeneous process likely related to partial alveolar collapse. The presence of such retained air spaces facilitates differentiation of disease severity, as more complete filling would suggest consolidation or advanced disease. Since MSCT was not performed on this specific specimen, its imaging conditions were simulated by down sampling the PBI-CT data and adjusting the slice thickness accordingly. As shown in Fig. 8B, the subtle parenchymal features and delineation of secondary pulmonary lobules were lost at the lower resolution, supporting the hypothesis that MSCT would likely have missed these findings. Histology (Fig. 8C) confirmed alveolar collapse, with thickened septa and disrupted architecture. Masson-Goldner staining (Fig. 8D) indicated connective tissue involvement.

### PBI-CT imaging of human FA-vapor fixed lungs

We examined two human right lungs preserved by FA vapor fixation, obtained from lung transplant recipients at Hannover Medical School: one with IPF and one with RA-associated UIP-pattern fibrosis. Although pre-imaging fixation of these human explants, necessary for biosafety and transport, differed from the porcine protocol, our previous study demonstrated that FA vapor fixation does not significantly impair PBI-CT image quality [16].

**Fig. 9:**
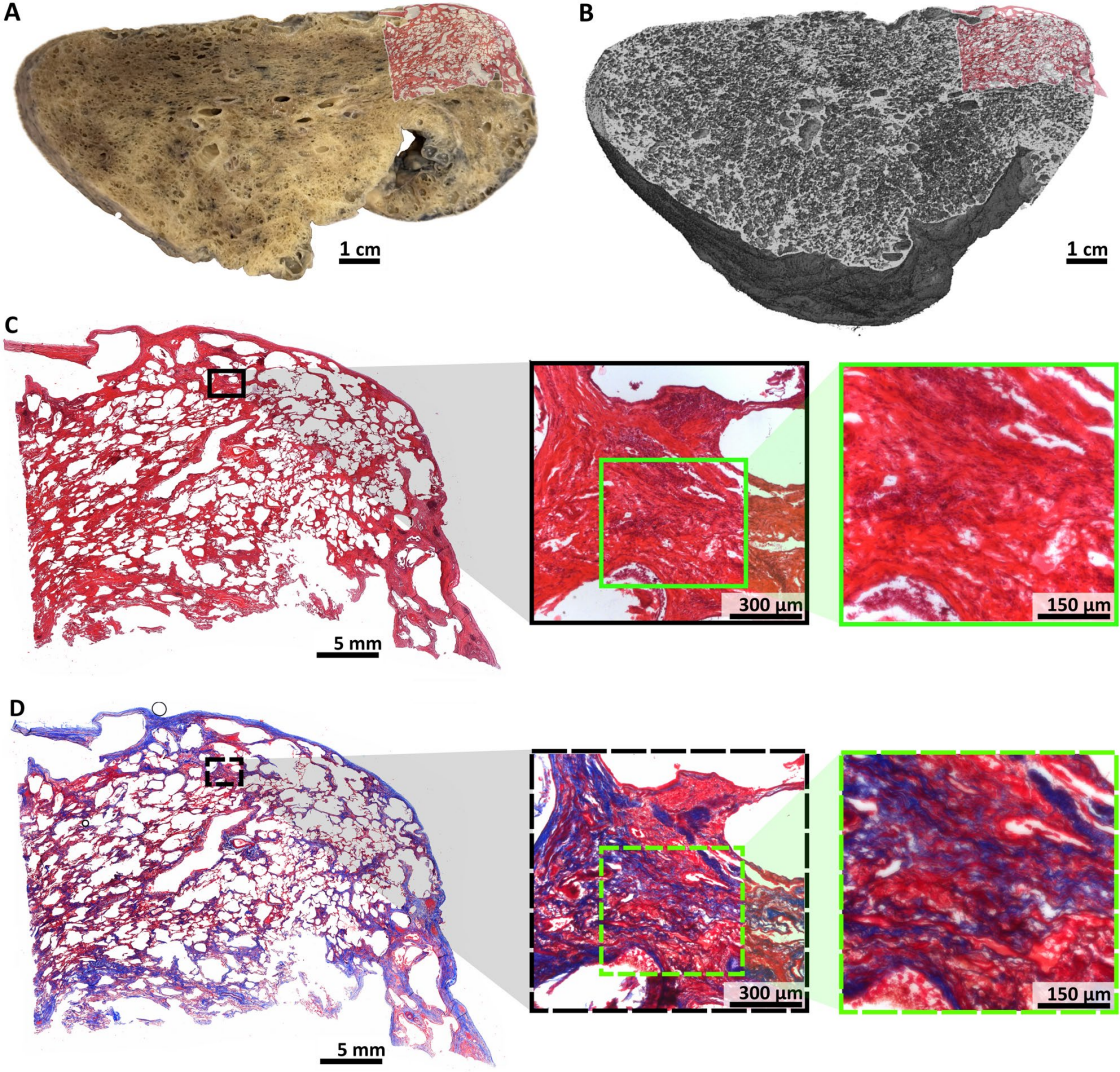
PBI-CT imaging of a human IPF lung, post-FA vapor fixation. **A** Shows a photo taken of a lamella of the FA vapor fixed right lung of an IPF patient. The lung appears dense and not fully inflated, potentially due to the high fiber content of the IPF lung. The subsequently performed H&E-stained histology is shown as an overlay. **B** 3D volume rendering of the PBI-CT data set (LAMBDA) of the same FA vapor fixed lung, shown in the same view as **A**. The subsequently performed H&E-stained histology is shown as an overlay. **C** H&E-stained section of the same lung region, shown in panels **A** and **B**. This histological analysis reveals that the lung tissue is almost entirely affected by a severe loss of acinar microarchitecture and thickening of the interalveolar septa, as seen in the higher magnification insets (black and green rectangles). **D** The corresponding Masson-Goldner stain reveals further histopathological detail. In contrast to Fig. 8, the thickened septa are intensely stained, clearly indicating that the septal thickening is caused by fibrosis and not microatelectasis, as seen in the higher magnification insets (black and green dashed rectangles). *PBI-CT* propagation-based synchrotron phase-contrast computed tomography, *IPF* idiopathic pulmonary fibrosis, *FA* formaldehyde *H&E* hematoxylin and eosin, *3D* three-dimensional

In the IPF lung (Fig. 9), PBI-CT revealed regions of preserved parenchymal structure despite partial collapse and extensive fibrosis (Fig. 9A, B). Notably, the anthropomorphic phantom’s design-imposed limitations on maintaining the physiological orientation of a single right lung. Corresponding histology (Fig. 9C) revealed severe loss of acinar microarchitecture and septal thickening, consistent with classical IPF features, while Masson-Goldner staining (Fig. 9D) confirmed collagen deposition.

**Fig. 10:**
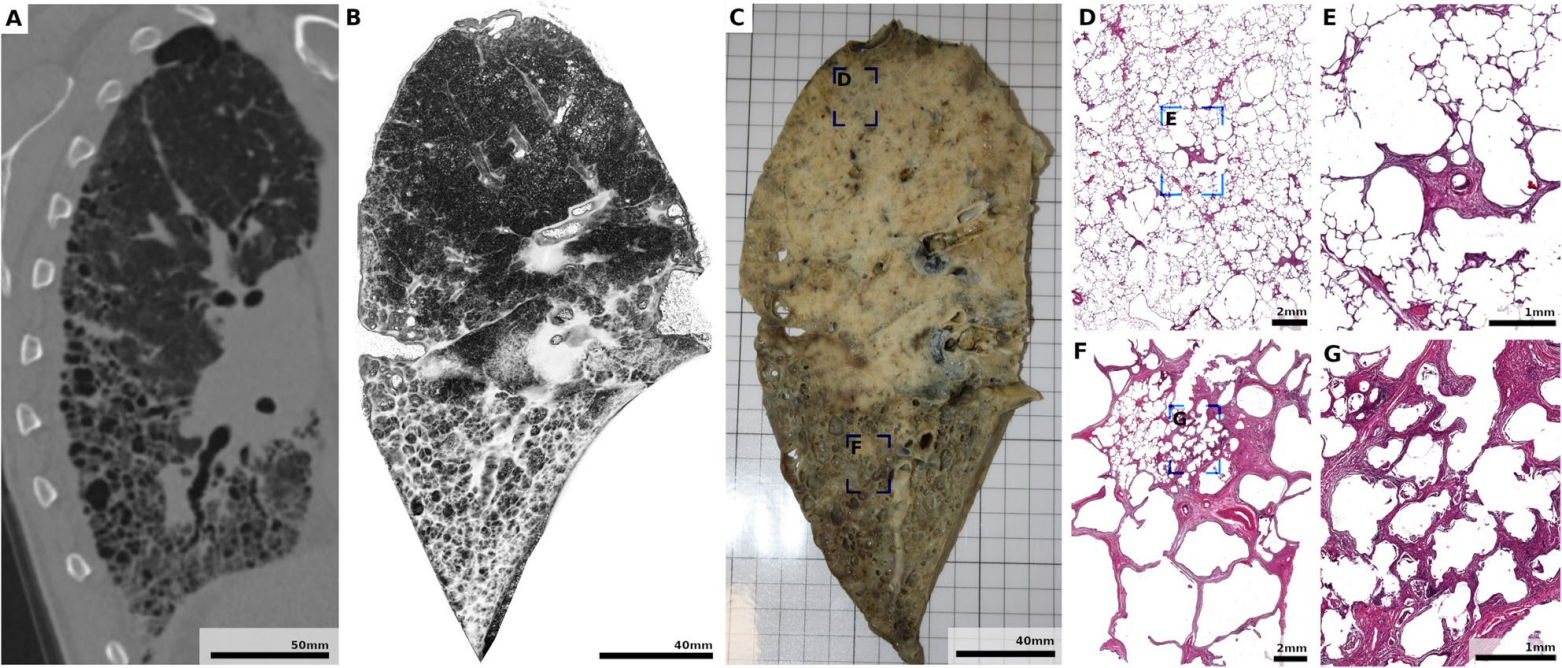
Radiologic–pathologic correlation of RA–associated UIP–pattern fibrosis. **A** In vivo MSCT acquired prior to lung transplantation, **B** ex vivo 3D volume rendering of the PBI-CT dataset (Hydra) obtained from the right lung within the phantom, after transplantation and subsequent FA vapor fixation, and **C** photograph of a lamella from the FA vapor-fixed lung, all shown in the same view. All modalities demonstrate extensive subpleural and basal-predominant reticulation with cystic airspaces and fibrotic remodeling, typical of a UIP-pattern fibrosis. Compared with in vivo MSCT, the ex vivo PBI-CT provides markedly higher spatial resolution and contrast, allowing visualization of fine cystic changes, fibrotic septa, and small airway alterations. Blue boxes in **C** indicate regions selected for histologic correlation. **D**–**G** H&E–stained sections from the indicated areas. **D**, **E** Relatively preserved parenchyma showing mild interstitial thickening and focal cystic dilatation of alveoli. **F**, **G** Severely fibrotic regions with cystic spaces, dense fibrotic walls, and architectural distortion consistent with end-stage remodeling. *MSCT* multislice computed tomography, *PBI-CT* propagation-based synchrotron phase-contrast computed tomography, *RA* rheumatoid arthritis, *UIP* usual interstitial pneumonia, *FA* formaldehyde, *H&E* hematoxylin and eosin, *3D* three-dimensional

In the RA-associated UIP-pattern fibrosis lung (Fig. 10), in vivo MSCT showed typical subpleural and basal-predominant reticulation (Fig. 10A), while ex vivo PBI-CT post FA vapor fixation in the phantom provided enhanced visualization of microstructural features, including fine cystic changes, fibrotic septa, and small airway remodeling, enabling a near-histologic assessment of fibrotic architecture in three dimensions (Fig. 10B). The spatial pattern of fibrosis closely matched the corresponding tissue lamella (Fig. 10C). Histologic correlation revealed a spectrum of remodeling: mild interstitial thickening and cystic alveolar dilation in preserved areas (Fig. 10D, E) versus dense fibrosis and architectural distortion in end-stage regions (Fig. 10F, G). Together, these results demonstrate that PBI-CT resolves pathological changes far beyond the capabilities of conventional MSCT, while FA vapor fixation preserves tissue architecture to enable precise radiologic–pathologic correlation.

While both human samples demonstrated technical feasibility, their advanced disease stages limited assessment of early or subtle alterations.

## Discussion

This study demonstrates the feasibility of ultra-high-resolution PBI-CT for imaging human-sized lungs within an anthropomorphic phantom at entrance radiation exposure levels comparable to full-dose MSCT (≈ 12 mGy). PBI-CT provides superior visualization of pulmonary microstructures, including peripheral airways and fine parenchymal details, revealing up to three additional airway generations beyond MSCT, as confirmed by radiological scoring and segmentation analyses.

To our knowledge, this is the first application of PBI-CT to human-sized lung specimens at entrance radiation dose levels equivalent to standard clinical imaging, with direct histological validation. Integration with FA vapor fixation preserved near-physiological tissue architecture and intrapulmonary air, enabling precise cross-modality correlation (MSCT, bronchoscopy, histology). Despite the logistical constraints that limited full multimodal correlation, including bronchoscopy, to a single porcine lung specimen, the implemented workflow is technically robust and can be reproducibly applied in future studies. Extending previous work in porcine models [11], we applied PBI-CT to FA-fixed human lungs with IPF and RA-associated UIP-pattern fibrosis, thereby demonstrating relevance to clinically important diseases.

Both photon-counting detectors, LAMBDA (55 × 55 µm^2^ nominal pixel size) and Hydra (100 × 100 µm^2^ nominal pixel size), delivered high image quality, outperforming MSCT. Reconstruction used conventional single-distance phase retrieval [19] followed by filtered back projection, and AI-based denoising (Noise2Noise). While denoising improved CNR, experts preferred the sharper, non-denoised images, highlighting a trade-off between noise suppression and spatial resolution. However, our results confirm that effective denoising can be achieved without high-dose reference scans. With ongoing advances in machine learning-based reconstruction [24], we anticipate further radiation dose reductions or enhanced spatial resolution in forthcoming applications. Resolution was mainly limited by the projected X-ray source spot size (≈ 200 μm), rather than detector pixel size. Upcoming synchrotron upgrades featuring smaller source spots will improve resolution and potentially allow lower radiation doses while maintaining diagnostic quality.

The ANTHONY phantom enables sequential, multimodal imaging, facilitating direct comparison of PBI-CT with MSCT, bronchoscopy, and histology. PBI-CT resolved peripheral airway structures beyond the reach of MSCT and bronchoscopy, offering non-invasive assessment of lung regions usually accessible only via transthoracic biopsy [25]. Histological validation confirmed PBI-CT distinguishes subtle tissue alterations and discriminates causes of “ground-glass opacities”, suggesting greater specificity than MSCT. We foresee its potential extending beyond fibrotic diseases to oncological applications, including early tumor detection, tumor microenvironment characterization, and identification of spread through air spaces (STAS).

While the observed imaging quality of post-mortem lung specimens is excellent, challenges remain for the final in vivo translation. Physiological lung motion from respiration and cardiac activity may introduce blurring, necessitating motion-robust imaging protocols and accelerated acquisitions. The limited FOV in PBI-CT, partly dependent on detector size, is not capable of whole-lung coverage, requiring strategic target localization via low-dose CT, with an associated radiation dose trade-off. In this study, scan times per rotation were 11.25 s (LAMBDA) and 36.00 s (Hydra) in step-and-shoot mode with 2.0–2.5 mm vertical FOV, requiring multiple vertical scans and stitching for full lung coverage. At the new SYRMEP-LS beamline, scan times of ≈ 10 s per rotation are anticipated, fast enough for most patients to hold their breath, enabling suppression of respiratory motion and reduction of motion artifacts. While the new beamline will offer an increased vertical FOV, multiple vertical scans are expected to remain necessary to ensure adequate anatomical coverage. Although slower than clinical MSCT (1.80 s for full lung coverage in this study), PBI-CT offers markedly higher spatial resolution and phase-contrast sensitivity, enabling visualization of pulmonary microstructures beyond the reach of MSCT. Thus, the temporal disadvantage is balanced by qualitative gains in soft tissue contrast and image detail. Large data volumes will, however, continue to pose challenges for storage, transfer, and analysis, necessitating advances in data management and intelligent analytics.

A limitation of this study is that the human lung specimens investigated represent advanced-stage disease (IPF and RA-associated UIP-pattern fibrosis) obtained at the time of lung transplantation. Consequently, these explants are not suitable for assessing the diagnostic sensitivity of PBI-CT for early or subtle disease manifestations, nor for extrapolating performance to healthy lung tissue. Advanced fibrosis is characterized by extensive architectural distortion, increased tissue density, and partial loss of alveolar air content. These changes may enhance the conspicuity of pathological features and thus overestimate detectability compared with early-stage disease or normal lungs. Therefore, the present human data primarily demonstrate technical feasibility, robustness of image quality, and the capability of PBI-CT to resolve complex pathological microstructures rather than its value for early diagnosis. In addition, in vivo patient CT data was obtained several weeks before lung transplantation and might therefore not represent the final pathological condition of the explants, limiting the comparison between clinical CT and PBI-CT. Importantly, the porcine lungs, imaged fresh, inflated, and largely free of overt pathology, provide a closer approximation to healthy lung tissue and support the method’s ability to visualize fine microanatomical structures under near-physiological conditions. Future studies will need to include human lungs with preserved or mildly altered architecture, as well as in vivo patient imaging, to determine the true diagnostic gain of PBI-CT in early disease detection and to establish its applicability across the full spectrum of lung health and disease.

Overall, phase sensitive X-ray imaging techniques have gained attention for lung imaging in ex vivo specimens [26], small animal models [27–29], and recent dark-field trials for emphysema [30], chronic obstructive pulmonary disease [31], and coronavirus disease 2019 [32]. Dark-field imaging is compatible with conventional X-ray sources and clinical environments, but currently limited to 2D. Synchrotron-based PBI-CT, while resource-intensive and limited in availability, provides unparalleled imaging quality, motivating clinical programs at facilities such as Elettra (Italy) and IMBL (Australia), where dedicated patient imaging initiatives are underway.

## Conclusion

In summary, PBI-CT represents a powerful complement to MSCT, delivering noninvasive, high-resolution pulmonary microanatomy imaging at clinically acceptable radiation doses. It holds promise as a second-level diagnostic tool for cases where MSCT results are inconclusive, potentially reducing invasive biopsy rates. Future progress in synchrotron source technology, motion compensation, detector design, and machine learning reconstruction will be critical to transition PBI-CT from proof-of-concept into clinical practice, ultimately advancing early diagnosis and monitoring of lung diseases.

## Supplementary Information

Supplementary Material 1.

## Data Availability

Due to the exceptionally large size of the PBI-CT data sets, public hosting on an open-access web service is not feasible. However, the data are available from the corresponding authors upon reasonable request.

## Abbreviations

2D: Two-dimensional
3D: Three-dimensional
AI: Artificial intelligence
ANTHONY: ANThropomorphic pHantom fOr luNg tomographY
CNR: Contrast-to-noise ratio
CT: Computed tomography
FA: Formaldehyde
FFPE: Formalin-fixed and paraffin-embedded
FOV: Field of view
FWHM: Full width at half maximum
H&E: Hematoxylin and eosin
IPF: Idiopathic pulmonary fibrosis
MRI: Magnetic resonance imaging
MSCT: Multislice computed tomography
PBI: Propagation-based imaging
PBI-CT: Propagation-based synchrotron phase-contrast computed tomography
RA: Rheumatoid arthritis
STAS: Spread through air spaces
STP: SYRMEP Tomo Project
SYRMEP: SYnchrotron Radiation for MEdical Physics
SYRMEP-LS: SYnchrotron Radiation for MEdical Physics - Life Science
TLD: Thermoluminescence detector
UIP: Usual interstitial pneumonia
